# Observations of TCRV**β** Gene Expression in Rats with Dampness Syndrome

**DOI:** 10.1155/2014/373608

**Published:** 2014-05-15

**Authors:** Yan Chen, Bao-Guo Sun, Shi-Jun Zhang, Ze-Xiong Chen, Carlini Fan Hardi, Ting Xiang

**Affiliations:** Department of Traditional Chinese Medicine,The First Affiliated Hospital of Sun Yat-Sen University, Guangzhou 510080, China

## Abstract

Environmental dampness is one factor which can cause human diseases. The effects of exposure to humidity on human immune function are diverse and numerous. In the theory of traditional Chinese medicine (TCM), dampness is defined as one of the major pathogenic factors in the human body. It is divided into “external dampness” and “internal dampness.” However the molecular mechanism leading to humidity-induced immunosuppression is obscure. In the present study, we investigated the expression of the T-cell antigen receptor variable *β* (TCRV*β*) subfamilies in rats which were fed in different humid environment. And the expression levels of the TCRV*β* subfamilies were detected using FQ-PCR. We found that the dampness might reduce the immunological recognition function of rats. And the obstruction of the immunological recognition function might be caused by internal dampness rather than external dampness.

## 1. Introduction

Environmental dampness has widespread effects on health [1–3]. Dampness is divided into “external dampness” and “internal dampness” in traditional Chinese medicine (TCM). External dampness is induced in many cases by environmental dampness, whereas internal dampness refers to metabolic imbalance caused by the dysfunction of liquid and humor. The associations between environmental dampness and a number of diseases have been identified in several epidemiological studies.

The effects of exposure to humidity on immune function are diverse and numerous, and several studies have shown that stress resulting from humidity is associated with suppression of several T-cell functions and a defective immune response [4–6].

The molecular mechanism leading to humidity-induced immunosuppression is obscure, and the aim of this study was to elucidate the possible effects of environmental dampness on the T-cell antigen receptor variable *β* (TCRV*β*) among rodents.

## 2. Materials and Methods

### 2.1. Animals

Female Sprague Dawley (SD) rats, weighing 230 ± 10 g, were obtained from the Animal Experiment Center of Sun Yat-Sen University. The procedures were performed according to the Institutional Guidelines for the Care and Use of Laboratory Animals. The rats were randomly housed in groups of four per wire-mesh cage (39 × 26 × 21 cm) for at least 1 week.

### 2.2. Experimental Design

A total of 32 SD rats were randomly divided into four groups with eight rats in each group as follows.

In Group I (normal group) the rats were placed in a controlled environment of 22 ± 1°C and 55% ± 5% relative humidity with free access to standard pelletized food (designed and supplied by the Animal Experiment Center of Sun Yat-Sen University) and tap water, and they were subjected to a 12-hour light/dark cycle.

In Group II (external dampness group) the rats were placed in a controlled environment of 26 ± 1°C and 94 ± 5% relative humidity with free access to standard pelletized food (designed and supplied by the Animal Experiment Center of Sun Yat-Sen University) and tap water, and they were subjected to a 12-hour light/dark cycle. We let the rats swim in a 20 cm deep pool with a temperature between 25°C and 27°C for 15 minutes every day.

In Group III (internal dampness group) the rats were placed in a controlled environment of 23 ± 1°C and 55 ± 5% relative humidity with free access to pelletized food with high glucose and fat contents (designed and supplied by the Animal Experiment Center of Sun Yat-Sen University) and 2 mL of frozen water intragastrically once every day, and they were subjected to a 12-hour light/dark cycle.

In Group IV (internal and external dampness group) the rats were placed in a controlled environment of 26 ± 1°C and 94 ± 5% relative humidity with free access to pelletized food with high glucose and fat contents (designed and supplied by the Animal Experiment Center of Sun Yat-Sen University) and 2 mL of frozen water, and they were subjected to a 12-hour light/dark cycle. We allowed the rats to swim in a 20 cm deep pool with a temperature between 25°C and 27°C for 15 minutes every day.

The observation period was 20 days. Three rats were randomly selected from each group to examine the expression levels of the TCRV*β* subfamilies.

### 2.3. Dampness Syndrome Criteria [7]

- Apathy, persistent lying down, and lazy appearance (staying together);
- appetite loss;
- loose stool;
- reduced drinking.

The animal models showed symptoms analogous to those of humans such as fatigue, loss of appetite, change of fur color, and weight loss.

### 2.4. The Detection of TCRV*β* Subfamilies Expression

#### 2.4.1. Extraction of the Splenocytes

We prepared a lymphocyte suspension and added 5 mL of Lympholyte-M to the centrifuge tube. Using a pipette, we carefully layered 5 mL of the cell suspension over the Lympholyte-M with as little mixing as possible at the interface, followed by centrifugation for 20 minutes at 1000–1500 G at room temperature. After centrifugation, there was a well-defined lymphocyte layer at the interface. Using a Pasteur pipette, we carefully removed the cells from the interface and transferred them to a new centrifuge tube. We diluted the isolated cells with medium and then centrifuged for 10 minutes at 800 G. We discarded the supernatant and washed the lymphocytes 2-3 times in the medium before further processing.

#### 2.4.2. Extraction of the Total Cellular RNA

The total cellular RNA was extracted from the spleen via acidic guanidinium thiocyanate-phenol-chloroform extraction according to the directions in the Trizol Kit (Gibco BRL, Gaithersburg, Maryland, USA), reverse transcribed into the first single-strand cDNA using a random hexamer primer and reverse transcriptase Superscript II Kit (Gibco BRL, Gaithersburg, Maryland, USA) according to the manufacturer's instruction, and stored at −80°C.

#### 2.4.3. Primer Design and Synthesis [8]

The primers were synthesized on an applied biosystems DNA synthesizer (Shanghai Shenggong Company, Shanghai, China), and all of the sequences have been published previously.

#### 2.4.4. Fluorescence Quantitative PCR (FQ-PCR)

The FQ-PCR analysis was performed using the SYBR green PCR system (Applied Biosystems, Foster City, CA) on a PE 7000 sequence detector (Perkin Elmer, Waltham, Massachusetts, USA). All of the PCR reactions were performed in triplicate. The real-time PCR efficiencies for each reaction were calculated using the following formula: Efficiency (*E*) = [10(1/slope)] − 1, from the slope values given in the PE 7000 sequence detector system. Real-time fluorescence measurements were taken, and a threshold cycle (CT) value for each sample was calculated by determining the point at which the fluorescence intensity exceeded a threshold limit (10 times the standard deviation of the baseline) by a model 7000 Sequence Detector. The CT values for the TCRV*β* transcripts from the specimens were plotted on the standard curve, and the amounts (fg) of TCRV*β* transcripts were calculated automatically using Sequence Detector version 1.6 (PE Applied Biosystems), a software package for data analysis. Real-time quantitative PCR of all of the samples was performed at the same time with the same well plate. Each sample was tested in duplicate, and the average of the two values was used for the calculation. The data were analyzed using LightCycler analysis software.

### 2.5. Determination of TCRV*β* Genes Usage

We obtained the data of TCRV*β* Genes Usage with the eqiupment of Automatic Fluorescence Quantitative PCR Instrument ABI 7500 (ABI Company, Carlsbad, California, USA). After the reaction, the results were analyzed and calculated automatically by a computer. Taking into account the various samples of the total RNA concentration differences, the ultimate results were in accordance with the following formula conversion: A (copies/*μ*g total RNA) = B (copies/*μ*L cDNA) ÷ OD260 × 1.05.

The percentage of each V*β* usage was calculated as the ratio of the relative amount of the specific V*β* gene expressed to the total amounts of the expression of 20 V*β* genes [8], as shown at Table 1.

### 2.6. Statistical Analyses

We analyzed the data with ANOVA, and the comparison between the two groups was achieved with the LSD test using SPSS 16 for Windows software.

## 3. Results

### 3.1. The Change in the Symptoms and Physical Signs

Except in the normal group, symptoms of dampness appeared in the rats of other groups approximately 5–7 days after the model-making process started; the most obvious symptoms were observed in the internal and external dampness group. During the experimental process, three deaths occurred in the internal dampness group, and five deaths occurred in the internal and external dampness group. Before death, the rats presented typical dampness symptoms, including joint swelling or erosion. No death occurred in the external dampness group or the normal group.

### 3.2. The Expression Level of the TCRV*β* Subfamilies

In the comparison of all of the groups, there were significant differences in the expression levels of TCRV*β*1, TCRV*β*7, TCRV*β*9, TCRV*β*13, and TCRV*β*18 in the spleen tissue of the rats (*P* < 0.001, *P* < 0.05, *P* < 0.05, *P* < 0.001, and *P* < 0.01, resp.).

The expression levels of TCRV*β*1, TCRV*β*7, TCRV*β*9, TCRV*β*10, and TCRV*β*13 in Group II were higher than those in Group I (*P* < 0.001, *P* < 0.05, *P* < 0.01, *P* < 0.05, and *P* < 0.001, resp.).

The expression levels of TCRV*β*1, TCRV*β*7, TCRV*β*13, and TCRV*β*18 in Group II were higher than those in Group III (*P* < 0.001, *P* < 0.05, *P* < 0.001, and *P* < 0.05, resp.), whereas the expression of TCRV*β*12 in Group II was lower than that in Group III (*P* < 0.05).

The expression levels of TCRV*β*1, TCRV*β*7, TCRV*β*9, TCRV*β*10, TCRV*β*13, and TCRV*β*18 in Group II were higher than those in Group IV (*P* < 0.001, *P* < 0.01, *P* < 0.05, *P* < 0.05, *P* < 0.001, and *P* < 0.01, resp.).

The expression levels of TCRV*β*8, TCRV*β*14, and TCRV*β*18 in Group III were lower than those in Group I (*P* < 0.05, *P* < 0.05, and *P* < 0.01, resp.); however, the expression of TCRV*β*9 in Group III was higher than that in Group I (*P* < 0.05).

The expression of TCRV*β*17 in Group III was higher than that in Group IV (*P* < 0.05).

The expression levels of TCRV*β*8 and TCRV*β*18 in Group IV were lower than those in Group I (*P* < 0.05 and *P* < 0.01, resp.); however, the expression of TCRV*β*19 in Group IV was higher than that in Group I (*P* < 0.05).

## 4. Discussion

According to TCM, health is largely dependent upon the continual transformation and movement of various energetic and material substances throughout the body. When the fluids in the body are prevented from being properly transformed and moved to their destination, dampness ensues.

Dampness is one of the major pathogenic factors in the human body as defined by Chinese medicine [9, 10]. The dampness of TCM includes “external dampness” and “internal dampness”. External dampness is induced in many cases by environmental factors, typically during damp weather or in situations in which a person comes into contact with moisture for extended periods of time, as with high humidity, sweat-drenched clothes, or wet or damp environments. Occupations such as gardening, working in a laundry and dishwashing might be risk factors for dampness diseases. Internal dampness is likely to occur from intake of excessive sweets, dairy products, starchy and glutinous foods, greasy or fried foods, watery fruits and vegetables, alcohol, and raw, cold, greasy, or sweetened food. Internal dampness is typically accompanied by weakness in the body's digestive system, which is responsible for separating food into nutrition and waste; a weak digestive system causes the excretion of nutritional elements and allows unhealthy substances to remain in the body. When a weak digestive system allows material that should be excreted to circulate, proper fluid movement is hampered, causing dampness. The symptoms associated with dampness are not immediately life threatening and do not attract sufficient attention, even from traditional doctors practicing Chinese medicine.

Several epidemiological studies have identified the association between environmental dampness and a number of diseases, such as allergic diseases, skin diseases, and respiratory diseases [1–3, 11–13]. Lifestyle factors, especially unhealthy eating patterns, might affect human health [14, 15]. The effect of exposure to humidity on immune function has been identified, and several studies have shown that humidity stress is associated with suppression of several T-cell functions and defective immune response [4–6, 16, 17]. The molecular mechanism leading to humidity-induced immunosuppression is obscure.

TCR is a molecule found on the surface of lymphocytes (or T cells); it is responsible for recognizing antigens bound to major histocompatibility complex (MHC) molecules. Engagement of the TCR with an antigen and MHC results in activation of its T lymphocyte through a series of biochemical events mediated by associated enzymes, coreceptors, and specialized accessory molecules. TCR V*β*T cells are the major subset of T cells [18, 19]. An analysis of T-cell receptor variable gene repertoires might provide important information about the immune response to pathogens or immunopathological mechanisms. Most recent studies of TCRV*β* expression focused only on simple pathogens or their metabolites. There have been few studies of TCRV*β* expression in several groups of pathogens; that is, dampness-related pathogens might contain, in addition to a group of pathogen-associated antigens or a molecular pattern, several groups of pathogen-associated antigens or additional molecular patterns.

In previous studies, we found that dampness syndrome was closely related with decreased T immune function, which might be caused by a disorder of the TCRV*β* repertoire expression in chronic hepatitis B patients [20–22].

We discovered, in a comparison of all of the groups in this study, that the expression of TCRV*β* subfamilies in the dampness groups was disordered, and the expression of TCRV*β*1, TCRV*β*7, TCRV*β*9, TCRV*β*13, and TCRV*β*18 in the spleen tissue of rats is significant. The expression levels of TCRV*β*1, TCRV*β*7, TCRV*β*9, TCRV*β*10, and TCRV*β*13 in the external dampness group were higher than those in the normal group, whereas the TCRV*β* expression in the internal dampness group and in the internal and external group was seldom higher than that in the normal group. We hypothesized that internal dampness could cause a worse effect on health than external dampness because three rats died in the internal dampness group, whereas five rats died with typical presentation of dampness symptoms in the internal and external dampness group during the experiment; no death occurred in the external dampness group and normal group.

Much of the TCRV*β* expression in the internal dampness group and in the internal and external group were lower than that in the external group, which indicated that the immunological recognition function might be suppressed in the internal dampness group and in the internal and external dampness group. Whether external dampness is easily recognized by TCRV*β* to produce effective immune response against related antigens or whether internal dampness could not easily be recognized by TCRV*β* to produce effective immune response against related antigens is unknown.

Death did not occur in the external dampness group, probably because of the shorter experimental time; the effect of external dampness for longer durations would be worthy of further study.

These findings suggest that the obstruction of the immunological recognition function of T cells might be caused by internal dampness rather than by external dampness. Further studies are needed to clarify the cause of the differences between internal dampness and external dampness.

## Figures and Tables

**Table 1:** The usage of TCRV*β* expression in all groups (*n* = 4, $\overset{-}{x}\pm S$).

V*β*	Group I	Group II	Group III	Group IV
V*β*1	2.50 × 10^−3^ ± 5.00 × 10^−4^	1.05 × 10^−2^ ± 1.26 × 10^−3^ ^c,e,h^	2.78 × 10^−3^ ± 2.06 × 10^−3^	6.00 × 10^−4^ ± 1.00 × 10^−4^
V*β*2	3.27 × 10^−4^ ± 9.02 × 10^−5^	4.01 × 10^−3^ ± 7.60 × 10^−4^	1.05 × 10^−2^ ± 1.71 × 10^−2^	1.31 × 10^−3^ ± 1.53 × 10^−3^
V*β*3	3.69 × 10^−3^ ± 8.00 × 10^−4^	3.73 × 10^−3^ ± 2.67 × 10^−3^	3.29 × 10^−3^ ± 4.36 × 10^−3^	5.86 × 10^−4^ ± 3.17 × 10^−4^
V*β*4	1.62 × 10^−3^ ± 5.41 × 10^−4^	9.07 × 10^−3^ ± 7.39 × 10^−3^	1.39 × 10^−3^ ± 8.31 × 10^−4^	8.84 × 10^−4^ ± 3.09 × 10^−4^
V*β*5	1.83 × 10^−3^ ± 2.50 × 10^−4^	9.72 × 10^−3^ ± 9.24 × 10^−3^	7.36 × 10^−3^ ± 5.73 × 10^−3^	1.24 × 10^−3^ ± 2.39 × 10^−4^
V*β*6	2.21 × 10^−1^ ± 2.01 × 10^−2^	2.71 × 10^−1^ ± 1.98 × 10^−1^	1.84 × 10^−1^ ± 8.98 × 10^−2^	7.56 × 10^−2^ ± 3.96 × 10^−2^
V*β*7	4.26 × 10^−5^ ± 2.51 × 10^−6^	1.70 × 10^−3^ ± 1.15 × 10^−3^ ^a,d,g^	3.00 × 10^−4^ ± 5.00 × 10^−5^	1.00 × 10^−4^ ± 5.70 × 10^−6^
V*β*8	1.70 × 10^−1^ ± 1.80 × 10^−2^	1.48 × 10^−1^ ± 3.21 × 10^−2^	8.11 × 10^−2^ ± 4.80 × 10^−2^ ^a^	7.20 × 10^−2^ ± 6.07 × 10^−2^ ^a^
V*β*9	2.09 × 10^−2^ ± 1.65 × 10^−3^	6.52 × 10^−2^ ± 1.97 × 10^−2^ ^b,f^	4.90 × 10^−2^ ± 5.77 × 10^−3^ ^a^	3.26 × 10^−2^ ± 1.39 × 10^−2^
V*β*10	2.00 × 10^−4^ ± 4.00 × 10^−5^	4.00 × 10^−3^ ± 3.54 × 10^−3^	9.00 × 10^−4^ ± 6.70 × 10^−4^	5.00 × 10^−4^ ± 1.90 × 10^−4^
V*β*11	3.45 × 10^−5^ ± 1.72 × 10^−5^	1.77 × 10^−3^ ± 1.61 × 10^−3^	1.33 × 10^−2^ ± 1.56 × 10^−2^	4.37 × 10^−3^ ± 3.91 × 10^−3^
V*β*12	1.99 × 10^−2^ ± 7.90 × 10^−4^	2.50 × 10^−3^ ± 1.70 × 10^−4^	7.89 × 10^−2^ ± 7.584 × 10^−2^	1.91 × 10^−2^ ± 9.64 × 10^−3^
V*β*13	1.53 × 10^−2^ ± 2.52 × 10^−3^	3.59 × 10^−2^ ± 4.39 × 10^−3^ ^c,e,h^	7.10 × 10^−3^ ± 4.20 × 10^−3^	9.60 × 10^−3^ ± 7.06 × 10^−3^
V*β*14	4.43 × 10^−1^ ± 7.64 × 10^−2^	1.40 × 10^−1^ ± 1.21 × 10^−1^	8.17 × 10^−2^ ± 7.81 × 10^−2^ ^a^	3.42 × 10^−1^ ± 3.33 × 10^−1^
V*β*15	2.00 × 10^−3^ ± 2.00 × 10^−3^	1.26 × 10^−2^ ± 1.50 × 10^−2^	2.47 × 10^−3^ ± 2.18 × 10^−3^	3.14 × 10^−3^ ± 7.91 × 10^−4^
V*β*16	6.56 × 10^−8^ ± 2.41 × 10^−8^	7.40 × 10^−8^ ± 5.00 × 10^−8^	4.49 × 10^−8^ ± 2.95 × 10^−8^	2.13 × 10^−8^ ± 2.44 × 10^−8^
V*β*17	4.00 × 10^−4^ ± 7.00 × 10^−5^	9.00 × 10^−4^ ± 2.80 × 10^−4^	1.60 × 10^−3^ ± 1.44 × 10^−3^	1.00 × 10^−4^ ± 7.00 × 10^−5^
V*β*18	1.43 × 10^−4^ ± 4.51 × 10^−5^	1.22 × 10^−4^ ± 4.80 × 10^−5^ ^d,f^	3.12 × 10^−5^ ± 2.00 × 10^−5^ ^b^	1.43 × 10^−5^ ± 1.00 × 10^−5^ ^b^
V*β*19	5.52 × 10^−2^ ± 6.01 × 10^−3^	1.84 × 10^−1^ ± 1.11 × 10^−1^	2.32 × 10^−1^ ± 2.06 × 10^−1^	4.73 × 10^−1^ ± 2.36 × 10^−1^ ^a^
V*β*20	4.28 × 10^−3^ ± 2.15 × 10^−4^	1.72 × 10^−2^ ± 2.24 × 10^−2^	4.79 × 10^−3^ ± 8.15 × 10^−3^	2.61 × 10^−4^ ± 8.45 × 10^−5^

Compared with Group I, ^a^ *P* < 0.05, ^b^ *P* < 0.01, and ^c^ *P* < 0.001; compared with Group III, ^d^ *P* < 0.05 and ^e^ *P* < 0.001; compared with Group IV, ^f^ *P* < 0.05, ^g^ *P* < 0.01, and ^h^ *P* < 0.001; Group I refers to the normal group, Group II refers to the external dampness group, Group III refers to the internal dampness group, and Group IV refers to the internal and external dampness group.
